# Phylogenetic analyses place the monotypic *Dryopolystichum* within Lomariopsidaceae

**DOI:** 10.3897/phytokeys.78.12040

**Published:** 2017-04-07

**Authors:** Cheng-Wei Chen, Michael Sundue, Li-Yaung Kuo, Wei-Chih Teng, Yao-Moan Huang

**Affiliations:** 1 Division of Silviculture, Taiwan Forestry Research Institute, 53 Nan-Hai Rd., Taipei 100, Taiwan; 2 The Pringle Herbarium, Department of Plant Biology, The University of Vermont, 27 Colchester Ave., Burlington, VT 05405, USA; 3 Institute of Ecology and Evolutionary Biology, National Taiwan University, No. 1, Sec. 4, Roosevelt Road, Taipei, 10617, Taiwan; 4 Natural photographer, 664, Hu-Shan Rd., Caotun Township, Nantou 54265, Taiwan

**Keywords:** Fern, morphology, Papua New Guinea, phylogeny, recircumscription, taxonomy, the Solomon Islands

## Abstract

The monotypic fern genus *Dryopolystichum* Copel. combines a unique assortment of characters that obscures its relationship to other ferns. Its thin-walled sporangium with a vertical and interrupted annulus, round sorus with peltate indusium, and petiole with several vascular bundles place it in suborder Polypodiineae, but more precise placement has eluded previous authors. Here we investigate its phylogenetic position using three plastid DNA markers, *rbcL*, *rps4-trnS*, and *trnL*-*F*, and a broad sampling of Polypodiineae. We also provide new data on *Dryopolystichum* including spore number counts, reproductive mode, spore SEM images, and chromosome counts. Our maximum-likelihood and Bayesian-inference phylogenetic analyses unambiguously place *Dryopolystichum* within Lomariopsidaceae, a position not previously suggested. *Dryopolystichum* was resolved as sister to a clade comprising *Dracoglossum* and *Lomariopsis*, with *Cyclopeltis* as sister to these, but clade support is not robust. All examined sporangia of *Dryopolystichum* produced 32 spores, and the chromosome number of sporophyte somatic cells is ca. 164. Flow cytometric results indicated that the genome size in the spore nuclei is approximately half the size of those from sporophyte leaf tissues, suggesting that *Dryopolystichum* reproduces sexually. Our findings render Lomariopsidaceae as one of the most morphologically heterogeneous fern families. A recircumscription is provided for both Lomariopsidaceae and *Dryopolystichum*, and selected characters are briefly discussed considering the newly generated data.

## Introduction

**Figure 1.**
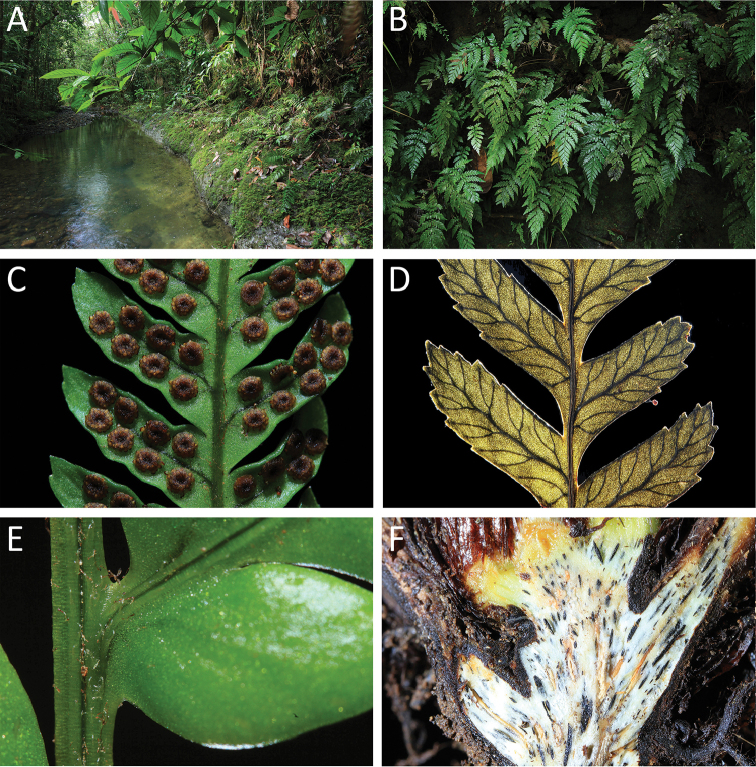
*Dryopolystichum phaeostigma* (based on *SITW10443*). **A** Habitat **B** Plants **C** Peltate indusia **D** Venation **E** Sulcate rachis-costa architecture **F** Longitudinal section of the rhizome.

*Dryopolystichum* Copel., with its single species *D. phaeostigma* (Ces.) Copel., is distributed along streams in lowland forests in New Guinea, the Bismarck Archipelago, and the Solomon Islands (Copeland 1947; Fig. 1A). Christensen (1937) was the first to point out that *D. phaeostigma* had been independently described under three different genera or subgenera. All told, generic placements has included *Aspidium* (≡ *Tectaria*) (Cesati 1877, Baker 1891), *Dryopteris* (Christensen 1906, Alderwerelt van Rosenburgh 1908, Copeland 1911, Brause 1920, Alderwerelt van Rosenburgh 1924), and *Polystichum* (Rosenstock 1911). Copeland (1947) inaugurated the new monotypic genus *Dryopolystichum* in his *Genera Filicum*, and argued that it was closest to *Ctenitis*. Pichi Sermolli (1977) agreed, citing the ctenitoid rachis, free venation, and peltate indusium as critical characters. Holttum included the genus in his “Tectarioid Group” in his list of Malaysian pteridophytes (Holttum 1959), but then omitted it in his 1991 treatment of that group.

Although Copeland did not provide an etymological explanation, the name *Dryopolystichum* presumably reflects the combination of peltate indusium (which is similar to those of polystichoid ferns) and pinnate-pinnatifid lamina division (which is similar to that of most *Dryopteris*). Such a combination of characters resulted in taxonomic confusion giving that peltate indusia are never found in *Dryopteris*, and the laminae of *Dryopolystichum* do not include prominulous segment apices, the hallmark of polystichoid ferns (Little and Barrington 2003). A peltate indusium is diagnostic of polystichoid ferns, including *Phanerophlebia* and *Polystichum*, but also found in a few distantly related genera in Polypodiineae such as *Cyclodium*, *Cyclopeltis*, *Rumohra*, *Megalastrum*, and *Tectaria* (Kramer and Green 1990).

Despite recent advances in fern phylogenetics and classification, the position of *Dryopolystichum* remains unclear. The thin-walled sporangium with a vertical and interrupted annulus, round sorus, and petiole with several vascular bundles suggest that this genus belongs to suborder Polypodiineae (= eupolypods I) (Sundue and Rothfels 2014, PPG I 2016). However, the remaining prominent features including pinnate-pinnatifid leaf dissection (Fig. 1B), peltate indusium (Fig. 1C), catadromous free veins (Fig. 1D), and sulcate rachis-costa architecture (Fig. 1E), do not clearly place it within any Polypodiinae family (Christensen 1937, Copeland 1947).

One other conspicuous character of *Dryopolystichum* not emphasized by previous authors is that the distal pinnae are decurrent onto the rachis, and the basal pinnules of its distal pinnae are served by veins that emerge from the rachis, rather than the pinna costa (Fig. 1D). This character is relatively uncommon in the Polypodiineae. It can be found in Dryopteridaceae, mostly in *Megalastrum*, and less commonly in *Stigmatopteris*, *Ctenitis*, and *Pleocnemia* (Moran et al. 2014, Moran and Labiak 2016). It can also be found in some Tectariaceae such as *Pteridrys* and *Tectaria* (Ding et al. 2014). Among these genera, *Pleocnemia* seems morphologically the most similar to *Dryopolystichum* because its rachises are adaxially sulcate and narrowly winged laterally. *Pleocnemia*, however, lacks a peltate indusium (Holttum 1974).

Subsequent to its establishment as a new genus in *Genera Filicum* (Copeland 1947), and Sermolli’s (1977) contribution, no other substantial argument was made for generic placement of *Dryopolystichum*. More recent studies maintained *Dryopolystichum* as a distinct genus, placing it under Dryopteridaceae (Kramer and Green 1990, Smith et al. 2006, Christenhusz et al. 2011). The recently published community-derived classification for extant lycophytes and ferns also places *Dryopolystichum* in the Dryopteridaceae but without assigning it to subfamily (PPG I 2016).

To resolve the phylogenetic placement of *Dryopolystichum*, we employ a molecular phylogenetic approach using three chloroplast DNA regions, *rbcL*, *rps4-trnS*, and *trnL-F*. Based on our observations, we further provide new data on *Dryopolystichum* including spore counts, reproductive mode, spore SEM images, and a chromosome count. Finally, we discuss its diagnostic characters in the light of the inferred phylogeny.

## Materials and methods

We examined the morphology of *Dryopolystichum phaeostigma* using material collected from the Solomon Islands (*Braithwaite R.S.S.4557*, SING; *SITW10443*, BSIP, TAIF, TNM) and Papua New Guinea (*James & Sundue 1688*, BISH, LAE, VT).

Living plants of *SITW10443* were transplanted to the Dr. Cecilia Koo Botanic Conservation Center in Taiwan (KBCC). The collection of *SITW10443* was made under the “Census and Classification of Plant Resources in the Solomon Islands” project (http://siflora.nmns.edu.tw/). Mitotic chromosomes were counted from these cultivated plants following the protocol of Chen et al. (2014).

Fertile pinnae of *SITW10443* were air-dried in an envelope for one day to release the spores. The spores were observed and measured by a tabletop scanning electron microscope (TM-3000 Hitachi, Ibaraki, Japan). The sizes (the length of equatorial axes including the perine ornamentation) of 35 randomly selected spores were measured. Five intact sporangia were observed under a stereo microscope (Leica MZ6, Wetzlar, Germany) to count the number of spores per sporangium.

The genome sizes of spore and leaf nuclei of *SITW10443* were examined by flow cytometry in order to infer the reproductive mode (Kuo et al. 2017). The genome size of spore nuclei should be half the genome size of leaf nuclei in the case of sexual and the same size in the case of apomictic reproduction (Kuo et al. 2017). We followed Kuo et al. (2017) for the extraction of leaf nuclei. For extraction of spore nuclei, we used an optimized bead-vortexing treatment with vertex duration of 1 minute and vertex speed of 1,900 rpm, as described by Kuo et al. (2017). An external standard was not necessary since we only need to compare the two phases of the life-cycle to each other.

### DNA extraction, amplification and sequencing

Total DNA was extracted using a modified CTAB-Qiagen column protocol (Kuo 2015). Three plastid DNA regions, *rbcL*, *rps4-trnS* (*rps4* gene + *rps4-trnS* intergenic spacer), and *trnL-F* (*trnL* gene + *trnL-trnF* intergenic spacer), were amplified and sequenced using the primers “ESRBCL1F” and “1379R” for *rbcL* (Pryer et al. 2001, Schuettpelz and Pryer 2007), “RPS5F” and “TRNSR” for *rps4-trnS* (Nadot et al. 1995, Smith and Cranfill 2002), and “FernL 1Ir1” and “f” for *trnL-F* (Taberlet et al. 1991, Li et al. 2010).

The PCR amplifications were performed in 16 μl reactions containing ca. 10 ng template DNA, 1×Taq DNA Polymerase Master Mix RED solution (Ampliqon, Denmark), and 1 μl each of 10 μM primers. The PCR reactions were carried out in a GeneAmp PCR System 9700 (Applied Biosystems, Carlsbad, California, USA). Thermocycling conditions were the same for PCRs of these three regions and comprised an initial denaturation of 2 minutes at 94°C followed by a core sequence of 35 repetitions of 94°C for 1 minute, 55°C for 1 minute, and 72°C for 1 minute followed by a final extension of 10 minutes at 72°C. Resulting PCR products were sequenced using the same PCR primers with BigDye^TM^ terminator (Applied Biosystems, Carlsbad, California, USA). The newly generated sequences were deposited in GenBank. GenBank accession numbers and voucher information are provided in Appendix.

### DNA alignment and phylogenetic analyses

Initial BLAST against the NCBI nucleotide database (Altschul et al. 1990) based on *rbcL* sequences indicated that *Dryopolystichum phaeostigma* is closely related to the species of Polypodiineae families, including Lomariopsidaceae, Nephrolepidaceae, Tectariaceae, and Dryopteridaceae. Accordingly, we assembled a data matrix including 250 species representing 36 genera from these families (Appendix). Sampling included all the four genera in which *D. phaeostigma* has been placed (i.e., *Dryopteris*, *Polystichum*, and *Tectaria*).

Sequences were aligned using Geneious v6.1.8 (Drummond et al. 2011) and then manually checked for errors. The three single-region (*rbcL*, *rps4-trnS*, and *trnL-F*) and dataset combining all three were independently subjected to both maximum likelihood (ML) and Bayesian inference (BI) phylogenetic analyses. Data matrices are available in TreeBASE, study number 20506, at https://treebase.org/. ML tree searches were conducted using RAxML (Stamatakis 2006) employing the GTRGAMMA substitution model through the CIPRES portal (Miller et al. 2010). Five independent searches for the ‘best tree’ and 1,000 bootstrap replicates were performed using a region-partitioned dataset. BI analyses were conducted using MrBayes 3.2.1 (Ronquist and Huelsenbeck, 2003) employing the same substitution model as in ML analysis. Each analysis consisted of two independent runs with four chains for 10^6^ generations, sampling one tree every 1000 generations. Burn-in was set to 10000 based on our preliminary analysis. The convergences of MCMC runs were checked using Tracer v.1.6 (Rambaut et al. 2014).

We addressed the possibility of phylogenetic bias due to long branches following the recommendation of Siddal and Whiting (1999). Since *Dracoglossum* and *Lomariopsis* were resolved on long branches in preliminary analyses (not shown), we conducted two additional analyses in which each one of the two long-branched genera, *Dracoglossum* and *Lomariopsis*, was excluded to examine whether phylogenetic placement and branch support for *Dryopolystichum*’s placement changed. Since maximum parsimony (MP) phylogeny is considered to be more susceptible to long-branch attraction (Philippe et al. 2005), we analyzed the concatenated dataset under MP in order to compare those results with our ML phylogeny. The MP analyses were conducted using TNT (Goloboff et al. 2008) following the search strategy detailed in Sundue et al. (2014).

## Results

### Phylogenetic analyses

**Figure 2.**
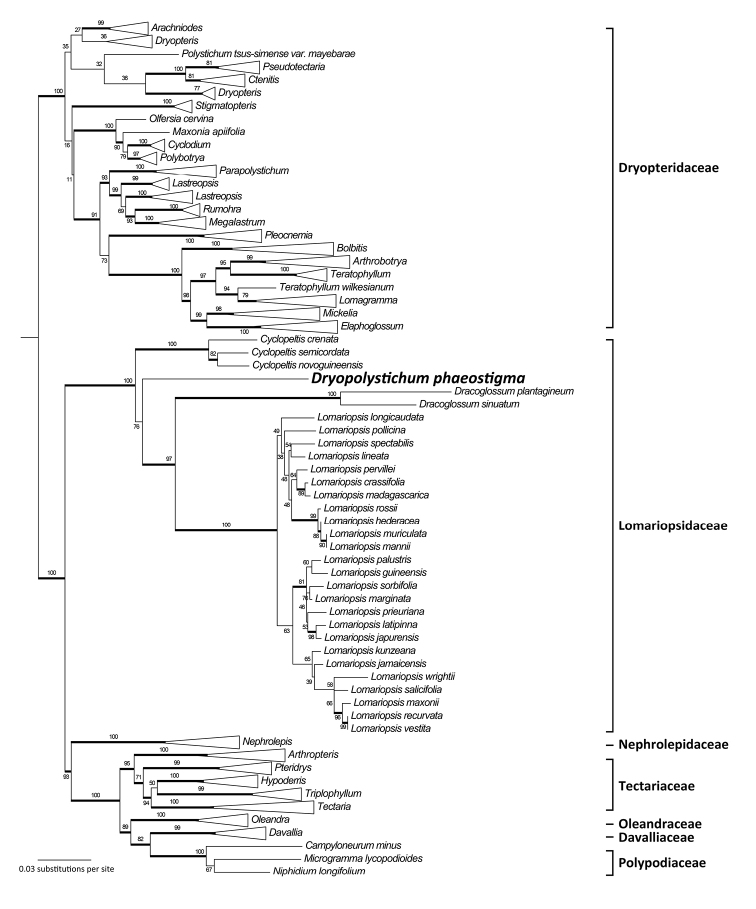
Simplified maximum likelihood phylogram of Polypodiineae obtained from the *rbcL* + *rps4-trnS* + *trnL-F* combined dataset. Maximum likelihood bootstrap percentages (BS) are provided at each node. Thickened lines indicate Bayesian inference posterior probability (PP) ≥ 0.9. Original phylogram with support values for all the nodes is available in Suppl. materials 1. Voucher information and GenBank accession numbers are shown in Appendix.

All single-region phylogenies resolved *Dryopolystichum phaeostigma* in Lomariopsidaceae, but with two slightly different topologies. The *rbcL* and *rps4-trnS* phylogenies placed *D. phaeostigma* sister to a clade of *Dracoglossum* + *Lomariopsis* with 93% and 72% maximum likelihood bootstrap percentages (BS), respectively (Suppl. materials 2, 3). In comparison, the *trnL-F* phylogeny placed *D. phaeostigma* sister to *Cyclopeltis* (BS = 74%), and *Dryopolystichum + Cyclopeltis* was sister to *Dracoglossum* + *Lomariopsis* (Suppl. material 4). There was no strongly supported conflict between the ML and BI phylogenies (Suppl. materials 1–4). Both the ML and BI phylogenies based on the combined dataset (Fig. 2, Suppl. material 1) reveal the same topology as those based on the *rbcL* and *rps4-trnS* regions. Bootstrap support and posteriori probability (PP) for the above relationships were generally very high except for the branches placing *D. phaeostigma*, where BS was ≤ 70% and PP were ≤ 0.9 in all the phylogenies.

Removing *Dracoglossum* from the analysis had little effect on the topology within Lomariopsidaceae, and BS supports for the generic placement of *Dryopolystichum* remained low (≤ 70%, data not shown). In contrast, the removal of *Lomariopsis* resulted in higher BS values for all clades within Lomariopsidaceae (≥ 99%, data not shown). MP analyses also resulted in a clade comprising all the Lomariopsidaceae genera and *Dryopolystichum*, but *Dryopolystichum* was resolved as sister to *Cyclopeltis* (data not shown).

### Karyology, reproductive mode, and spore measurements

**Figure 3.**
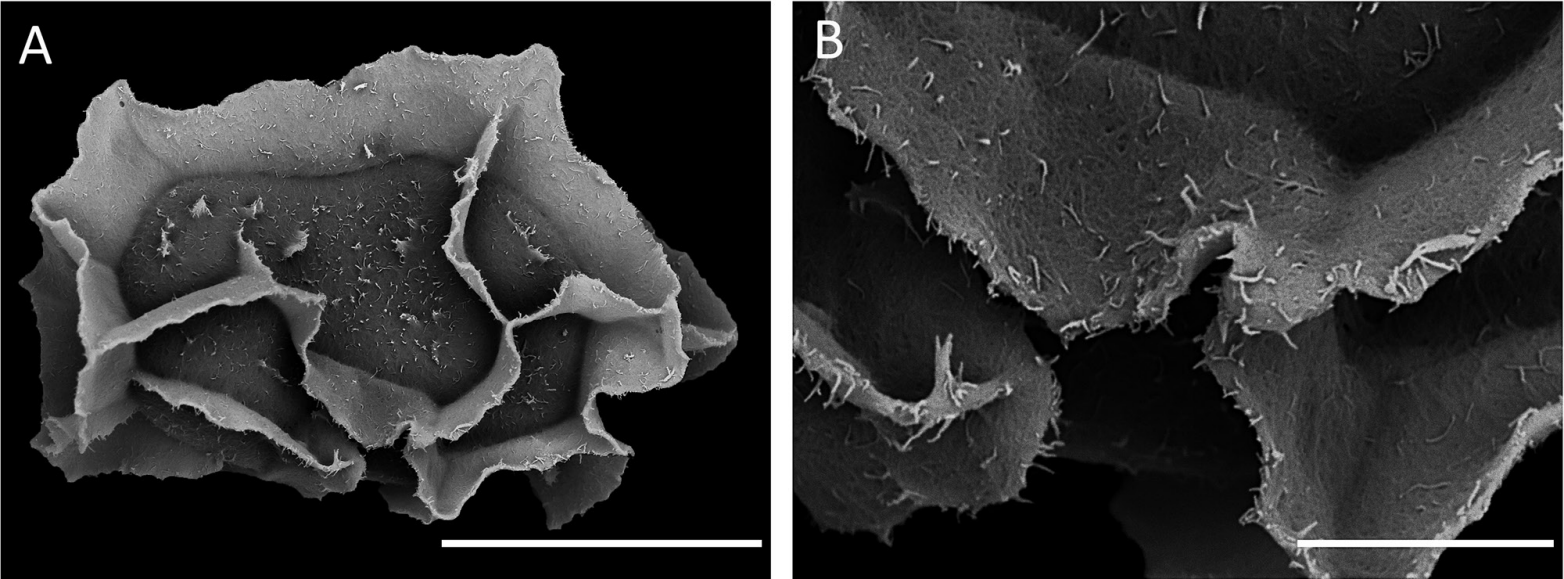
Spores SEM of *Dryopolystichum phaeostigma*. **A** Lateral view of the spore **B** Detail of surface. Scale bars: **A** = 50 μm, **B** = 10 μm.

**Figure 4.**
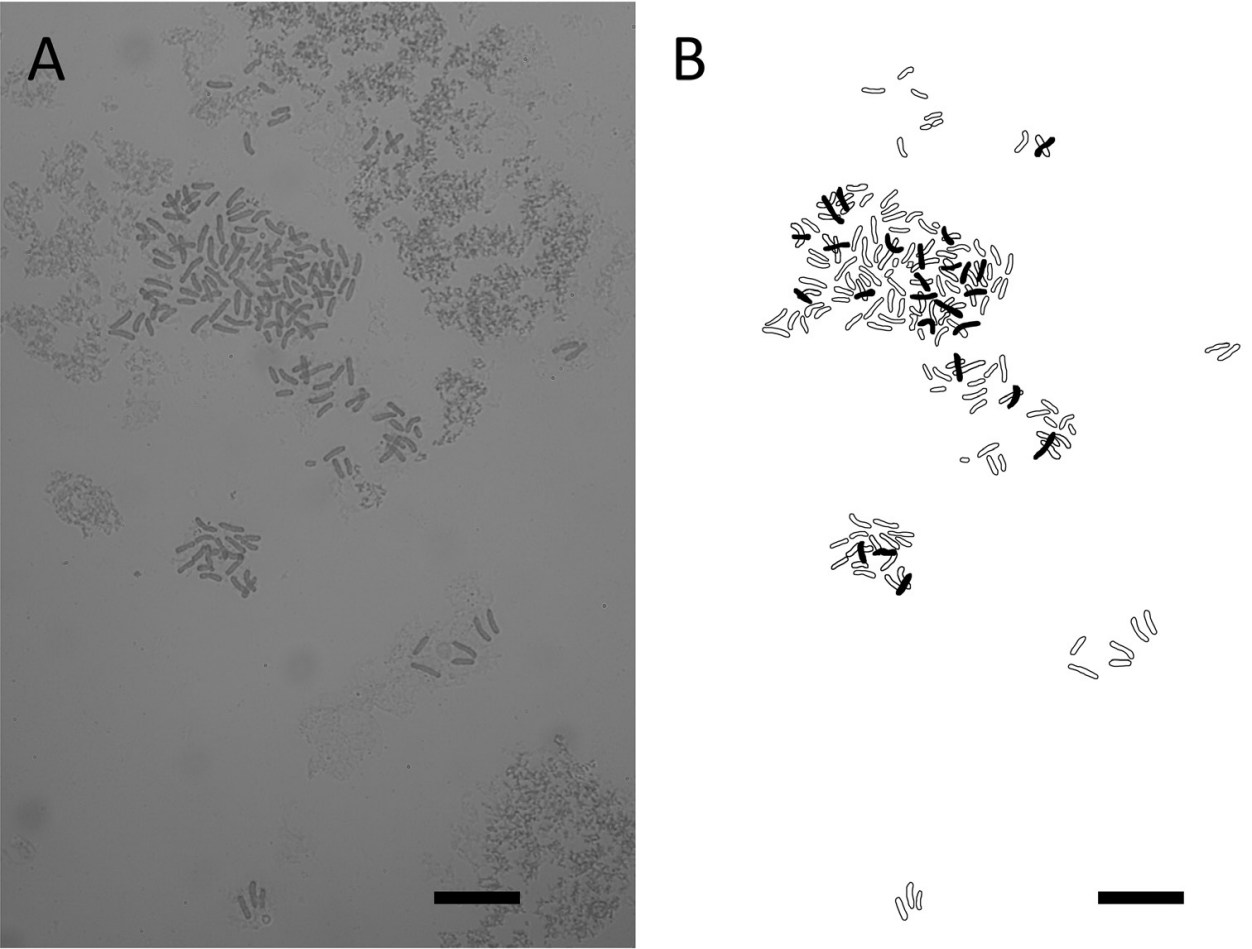
Chromosome number of *Dryopolystichum phaeostigma*. **A** Chromosomes at mitosis metaphase, 2n = ca. 164 (*SITW10443*) **B** explanatory illustration of A. Scale bars = 10 μm.

**Figure 5.**
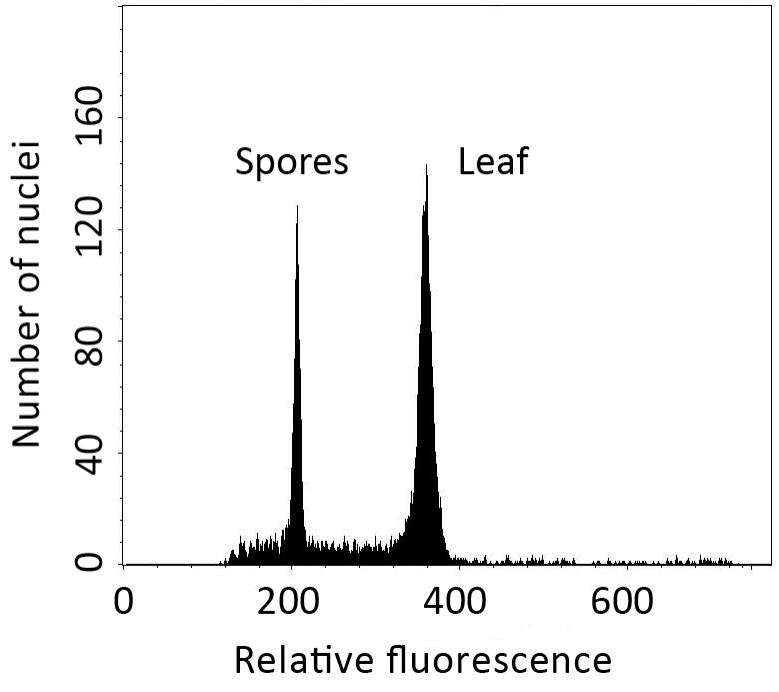
Relative DNA contents of *Dryopolystichum phaeostigma* spore and leaf nuclei inferred by flow cytometry.

All examined sporangia (*SITW10443*) produced 32 normal spores, and the mean spore length was 64.1 ± 4.5 μm (Fig. 3). The chromosome number of the three sporophyte somatic cells observed was ca. 164 (Fig. 4). Results of flow cytometry revealed that the genome size of spore nuclei is approximately half of those of leaf nuclei (Fig. 5).

## Discussion

### Phylogenetic placement of Dryopolystichum

The reconstructed maximum likelihood and Bayesian inference phylogenies unambiguously resolved *Dryopolystichum* within Lomariopsidaceae (Fig. 2), a position not previously suggested (Kramer and Green 1990, Smith et al. 2006, Christenhusz et al. 2011, PPG I 2016). This placement is consistent in all our analyses. Nonetheless, the generic position of *Dryopolystichum* within Lomariopsidaceae remains poorly resolved. This uncertainty may be partially explained by the incongruence between *trnL-F* and the other analyzed regions, but our process of removing the long-branched genera showed that low BS was retrieved only when *Dryopolystichum* and *Lomariopsis* were both included in the analysis. These results may also be explained by the large amounts of missing data in *Lomariopsis*; 19 of the 25 species included were represented by *trnL-F* data alone. We recommend further phylogenetic study using an expanded dataset to resolve the intergeneric relationships within Lomariopsidaceae.

### Recircumscription of Lomariopsidaceae

Phylogenetic analyses using DNA sequences have served as the basis for redrawing fern classifications in the 21^th^ century (Smith et al. 2006, Christenhusz et al. 2011, PPG I 2016). With respect to family circumscription, one of the most dramatically changed families is Lomariopsidaceae (Tsutsumi and Kato 2006, Schuettpelz and Pryer 2007, Christenhusz et al. 2013). Just prior to the molecular era, Lomariopsidaceae was treated as one of the largest fern families with six genera and over 500 species (e.g., Kramer and Green 1990) and was strongly supported by the following combination of characters: rhizomes with ventral root insertion, dictyosteles with elongate ventral meristeles, and dimorphic leaves where the fertile leaves had acrostichoid sori (Holttum and Hennipman 1959, Kramer and Green 1990).

Subsequent molecular phylogenetic analyses demonstrated that most genera previously treated in Lomariopsidaceae should be transferred to Dryopteridaceae (Tsutsumi and Kato 2006, Schuettpelz and Pryer 2007). The combination of characters uniting the former Lomariopsidaceae are now interpreted to have evolved multiple times, and to be correlated with dorsiventrality of the rhizome (Moran et al. 2010, McKeown et al. 2012). Meanwhile, *Cyclopeltis* was transferred from Dryopteridaceae to Lomariopsidaceae as suggested by molecular phylogeny (Schuettpelz and Pryer 2007), although it has none of the characters formerly used to circumscribe Lomariopsidaceae (Holttum and Hennipman 1959, Kramer and Green 1990).

More recently, the neotropical genus *Dracoglossum* was established (Christenhusz 2007) and later transferred to Lomariopsidaceae from Tectariaceae based on a molecular phylogeny (Christenhusz et al. 2013). This pattern was also unexpected since there are essentially no shared morphological characters by *Dracoglossum* and *Lomariopsis*, except for the ribbon-like gametophyte (R. C. Moran pers. com.). Our finding, that *Dryopolystichum* belongs to Lomariopsidaceae, comes as a further surprise. With these changes, Lomariopsidaceae is a family of five genera (*Cyclopeltis*, *Dracoglossum*, *Dryopolystichum*, *Lomariopsis*, and *Thysanosoria*) and ca. 70 species. As far as we can tell, none of the morphological traits commonly used unify these genera (Table 1). In the following paragraphs, we provide a recircumscription of both Lomariopsidaceae and *Dryopolystichum*, and then discuss selected characters in the light of our phylogenetic placement.

**Table 1.** Comparison of morphological characters of the five Lomariopsidaceae genera [based on Holttum and Hennipman (1959), Holttum (1991), Roubik and Moreno (1991), Moran (2000), Christenhusz (2007), Rouhan et al. (2007), and this study].

Genera	*Cyclopeltis*	*Dracoglossum*	*Dryopolystichum*	*Lomariopsis*	*Thysanosoria*
Habit	terrestrial	terrestrial	terrestrial	hemiepiphyte	hemiepiphyte
Rhizome	erect	short creeping	erect	climbing	climbing
Frond division*	pinnate	simple	pinnate-pinnatifid	pinnate	pinnate
Pinnae articulation	articulate	–	not articulate	articulate	articulate
Venation	free	reticulate, with included veinlet	free	free	free
Rachis-costa architecture	prominent	prominent	grooved	grooved or flat	grooved
Sporangia	form rounded sori	form rounded sori	form rounded sori	acrostichoid	form rounded sori
Indusia	peltate if present	peltate if present	peltate	absent	absent
Perine ornamentation	broad folds	narrow crests	narrow crests	various	broad folds

*matured plant, -not applicable

## Taxonomic treatment

### Lomariopsidaceae

Taxon classificationPlantaeORDOFAMILIA

Alston, Taxon 5(2): 25. 1956.

#### Type.

*Lomariopsis* Fée, Mém. Foug., 2. Hist. Acrostich.: 10. 1845.

#### Description.

Habit erect, creeping, or climbing; rhizomes dictyostelic, the ventral meristele elongate in transverse section or not; scaly at least when young; scales non-clathrate, basally attached or shallowly peltate, margins entire, toothed, or ciliate; fronds monomorphic or dimorphic; petioles with multiple vascular bundles arranged in a U-shape; laminae simple, pinnate, or pinnate-pinnatifid, provided distally with proliferous buds or not; pinnae articulate to the rachis or not; veins free, ± parallel or pinnate; sori acrostichoid or discrete and then round, with peltate indusia or exindusiate; spores brown, olive or green, chlorophyllous or not, bilateral, monolete, perine loosely attached, variously winged or ornamented.

Five genera and an estimated 70 species. *Thysanosoria* is included based on its morphological similarity to *Lomariopsis* (Holttum and Hennipman 1959), but it has not been, to the present, subject to molecular phylogenetic analysis.

### Dryopolystichum

Taxon classificationPlantaeORDOFAMILIA

Copel., Gen. Fil. 125, t. 4. 1947.

#### Type.

*Dryopolystichum phaeostigma* (Ces.) Copel., Gen. Fil. 125, t. 4. 1947.

#### Description.

Habit terrestrial, on slopes along streams at lowland forests; rhizome short erect, stout and woody, apex densely scaly, blackish sclerenchyma strands visible in sections; scales dark brown, linear-lanceolate, entire, not clathrate; fronds approximate, stipe not articulate, scaly at base, scales similar to those on rhizome; lamina ovate, pinnate-pinnatifid, catadromous, subleathery, nearly glabrous, only very sparse narrow scales on rachis, costa, and costule; rachis and costa grooved adaxially, not connected to each other; veins free, pinnate, veins of basal pinnules on upper pinnae emerge from the rachis rather than costa, all veins terminating in a prominent hydathode, not reaching frond margin; sori round, dorsally on veinlets near hydathode, indusiate; indusia round, persistent, superior, entire, brownish, thick; sporangia long-stalked, annulus with ca. 14 indurated cells, 32 normal spores in each sporangium; spores monolete, 64.1 ± 4.5 μm in lateral view, surface with broadly winged wall; 2n = ca. 164.

Monotypic.

### Dryopolystichum phaeostigma

Taxon classificationPlantaeORDOFAMILIA

(Ces.) Copel., Gen. Fil. 125, t. 4. 1947.

Aspidium phaeostigma Ces., Rend. Ac. Napoli 16: 26, 29. 1877. Type. Papua New Guinea. Andai, *Beccari 12533* (FI [FI013622]).
Dryopteris phaeostigma (Ces.) C.Chr., Index Filic. 284. 1905. Type. Based on Aspidium phaeostigma Ces.
Dryopteris tamatana C.Chr., Index Filic., Suppl. (1906-1912) 40. 1913. Replaced: Dryopteris kingii Copel., Phillipp. J. Sci., C 6: 73. 1911., not Dryopteris kingii (Bedd.) C.Chr., Index Filic. 273. 1905. Type. Papua New Guinea. Tamata, *C. King 149* (MICH [MICH1287049]).
Polystichum lastreoides Rosenst., Repert. Spec. Nov. Regni Veg. 9: 425. 1911. Type. Papua New Guinea. *C. King 194* (MICH [MICH1190927]).
Dryopteris ledermannii Brause, Bot. Jahrb. Syst. 56: 90. 1920. Type. Papua New Guinea. Sepik, *Ledermann 9619* (B [B_20_005865], L [L0063060], S [S-P-8581]).
Dryopteris cyclosorus Alderw., Nova Guinea 14: 21. 1924. Type. Indonesia. Irian Jaya, *H. J. Lam 1086* (BO [BO1529719, BO1529720], K [K000666126], L [L0051583], U [U0007385]).

#### Type.

Based on *Aspidium phaeostigma* Ces.

#### Description.

Equal to the genus.

#### Distribution.

New Guinea, the Bismark archipelago, and the Solomon Islands.

### Comparison of selected characters of Dryopolystichum

Perine architecture of *Dryopolystichum* is very similar to that of *Dracoglossum plantagineum* (Christenhusz 2007, Fig. 3). They are loosely attached, forming thin crests, and having a spiculate microstructure. Perine of *Cyclopeltis* and *Thysanosoria* are also similar in being loosely attached and having a spiculate microstructure, but they differ by having broader folds (Holttum and Hennipman 1959, Tryon and Lugardon 1991). The perine characters, however, are not shared by all the taxa of Lomariopsidaceae especially considering the variation of ornamentation existing in *Lomariopsis* (Rouhan et al. 2007). Moreover, these perine characters also appear in other Polypodiineae lineages particularly in bolbitidoid ferns (Moran et al. 2010) as well as in various Aspleniineae lineages (Sundue and Rothfels 2014, PPG I 2016).

Blackish sclerenchyma strands are visible in the rhizome sections of *Dryopolystichum* (Fig. 1F). These are also present in *Dracoglossum*, *Cyclopeltis*, and *Lomariopsis*, but similar characters are known from various groups throughout Polypodiineae (Hennipman 1977, Moran 1986, Hovenkamp 1998). Further studies might reveal variation in these strands to be of systematic value.

The rachis-costae architecture of *Dryopolystichum* is characterized by an adaxially sulcate rachis with grooves that do not connect to those of the pinna-costae. The rachis is also narrowly winged laterally. Both characters are seen in *Thysanosoria* and in some species of *Lomariopsis* (Holttum and Hennipman 1959, Moran 2000). In contrast, *Dracoglossum* and *Cyclopeltis* have non-winged and non-sulcate rachises (Holttum 1991, Christenhusz 2007).

The chromosome number in somatic cells of *Dryopolystichum phaeostigma* was ca. 164 (Fig. 4). The base numbers for Lomariopsidaceae genera (*Cyclopeltis*, *Dracoglossum*, and *Lomariopsis*) are 40 or 41 (Walker 1985, Kato and Nakato 1999, Moran 2000), suggesting that *D. phaeostigma* is a tetraploid.

Our flow cytometry and spore count results indicate that *Dryopolystichum phaeostigma* is sexually reproducing and has 32 spores per sporangium (Fig. 5). In Polypodiales, sporogenesis leading to the formation of 64 spores in a sporangium is by far the most common pattern of sexually reproducing species, e.g., Aspleniaceae (Gabancho et al. 2010), Athyriaceae (Kato et al. 1992, Takamiya et al. 1999), Davalliaceae (Chen et al. 2014), Dryopteridaceae (Lu et al. 2006), Polypodiaceae (Wang et al. 2011), Pteridaceae (Huang et al. 2006), and Thelypteridaceae (Ebihara et al. 2014). Cases of sporogenesis resulting in 32 spores per sporangium are known from a few Polypodiales ferns but all belong to the suborders Lindsaeineae and Pteridineae, i.e., Lindsaeaceae (Lin et al. 1990), Cystodiaceae (Gastony 1981), and *Ceratopteris* (Pteridaceae; Lloyd 1973). Our study provides the first confirmed case of a sexual reproduction with 32 spores per sporangium in the suborder Polypodiineae.

## Conclusion

We have shown, based on molecular phylogenetic evidence, the placement of *Dryopolystichum* within Lomariopsidaceae. A revised description was provided for both Lomariopsidaceae and *Dryopolystichum* resulting from a review of literature and our own observations. Future studies using an expanded dataset are necessary to resolve intergeneric relationships in Lomariopsidaceae.

## Supplementary Material

XML Treatment for Lomariopsidaceae

XML Treatment for Dryopolystichum

XML Treatment for Dryopolystichum phaeostigma

## Appendix

Individuals sampled in this study. For each individual, the species name and GenBank accession numbers (*rbcL*, *rps4-trnS*, *trnL-F*) are provided. A n-dash (–) indicates unavailable information; new sequences are in bold.

**Table:**

Taxon	Genbank accession numbers		
	*rbcL*	*rps4-trnS*	*trnL-F*
Dryopteridaceae			
*Arachniodes aristata* (G.Forst.) Tindale	KJ464418	–	KJ464592
*Arachniodes denticulata* (Sw.) Ching	KJ464419	–	KJ464593
*Arthrobotrya articulata* J.Sm.	–	GU376714	GU376565
*Arthrobotrya wilkesiana* Copel.	–	GU376719	GU376569
*Bolbitis acrostichoides* (Afzel.) Ching	KJ464420	GU376644	GU376500
*Bolbitis aliena* (Sw.) Alston	–	GU376646	GU376502
*Bolbitis angustipinna* (Hayata) H.Ito	–	GU376654	GU376509
*Bolbitis appendiculata* (Willd.) K.Iwats.	–	GU376647	GU376503
*Bolbitis auriculata* (Lam.) Alston	KJ464421	GU376649	GU376505
*Bolbitis bipinnatifida* (J.Sm.) K.Iwats.	–	GU376676	GU376530
*Bolbitis fluviatilis* (Hook.) Ching	–	GU376656	GU376510
*Bolbitis gemmifera* (Hieron.) C.Chr.	–	GU376657	GU376511
*Bolbitis heteroclita* (Pr.) Ching	–	GU376659	GU376513
*Bolbitis heudelotii* (Bory) Alston	–	GU376662	GU376515
*Bolbitis humblotii* (Baker) Ching	KJ464422	GU376663	GU376516
*Bolbitis lonchophora* (Kunze) C.Chr.	–	GU376664	GU376517
*Bolbitis major* (Bedd.) Hennipman	–	GU376665	GU376518
*Bolbitis portoricensis* (Sprengel) Hennipman	–	GU376670	GU376523
*Bolbitis salicina* (Hook.) Ching	–	GU376671	GU376525
*Bolbitis semipinnatifida* (Fée) Alston	–	GU376672	GU376526
*Bolbitis serratifolia* (Mertens) Schott	–	GU376673	GU376527
*Bolbitis sinuata* (C.Presl) Hennipman	–	GU376675	GU376529
*Bolbitis tibetica* Ching & S.K.Wu	–	GU376677	GU376531
*Ctenitis eatonii* (Baker) Ching	KF709483	–	KJ196645
*Ctenitis sinii* (Ching) Ohwi	–	–	KJ196643
*Ctenitis subglandulosa* (Hance) Ching	–	–	KJ196655
*Ctenitis yunnanensis* Ching & Chu H.Wang	–	–	KJ196715
Cyclodium heterodon (Schrad.) T. Moore var. heterodon	KJ464425	–	KJ464596
*Cyclodium rheophilum* A.R.Sm.	KJ464426	–	KJ464597
*Dryopteris apiciflora* (Wall. ex Mett.) Kuntze	–	–	KJ196641
*Dryopteris christensenae* (Ching) Li Bing Zhang	–	–	KJ196679
*Dryopteris heterolaena* C.Chr.	–	–	KJ196623
*Dryopteris integriloba* C.Chr.	–	–	KJ196701
*Dryopteris mariformis* Rosenst.	–	–	KJ196686
*Dryopteris nidus* (Baker) Li Bing Zhang	–	–	KJ196687
*Dryopteris patula* (Sw.) Underw.	KJ464427	–	KJ464598
*Dryopteris polita* Rosenst.	–	–	KJ196700
*Dryopteris squamiseta* (Hook.) Kuntze	–	GU376678	KJ196632
*Dryopteris wallichiana* (Spreng.) Hyl.	KJ464428	GU376680	KJ464599
*Elaphoglossum amygdalifolium* (Mett.) Christ	–	GU376681	–
*Elaphoglossum burchellii* (Baker) C.Chr.	–	GU376682	GU376533
*Elaphoglossum decoratum* (Kunze) T.Moore	KJ464429	GU376683	KJ464600
*Elaphoglossum guentheri* Rosenst.	–	GU376684	GU376535
*Elaphoglossum langsdorffii* T.Moore	–	GU376685	GU376536
*Elaphoglossum lloense* (Hook.) T.Moore	–	GU376686	GU376537
*Elaphoglossum luridum* Christ	–	–	GU376538
*Elaphoglossum squamipes* (Hook.) T.Moore	–	–	GU376539
*Lastreopsis amplissima* (C.Presl) Tindale	KJ464432	–	KJ464604
*Lastreopsis decomposita* (R.Br.) Tindale	KJ464439	–	–
*Lastreopsis hispida* (Sw.) Tindale	KJ464446	–	KJ464614
*Lastreopsis killipii* (C.Chr. & Maxon) Tindale	KJ464448	KF709505	–
*Lastreopsis marginans* (F.Muell.) Tindale	KJ464449	GU376691	KJ464616
*Lastreopsis poecilophlebia* (Hook.) Labiak, Sundue & R.C.Moran	KJ464423	GU376692	KJ464594
*Lastreopsis tenera* (R.Br.) Tindale	KJ464467	GU376699	KJ464636
*Lastreopsis tripinnata* (F.Muell. ex Benth.) Labiak, Sundue & R.C.Moran	KJ464491	GU376700	–
*Lastreopsis walleri* Tindale	KJ464472	GU376701	–
*Lastreopsis wurunuran* (Domin) Tindale	KJ464474	GU376704	–
*Lomagramma brooksii* Copel.	–	GU376705	GU376542
*Lomagramma cordipinna* Holttum	–	GU376707	GU376543
*Lomagramma lomarioides* (Blume) J.Sm.	–	–	GU376550
*Lomagramma matthewii* (Ching) Holttum	KJ464476	–	KJ464640
*Lomagramma perakensis* Bedd.	–	–	GU376552
*Lomagramma pteroides* J.Sm.	–	–	GU376555
*Lomagramma sinuata* C.Chr.	–	–	GU376556
*Lomagramma sumatrana* Alderw.	–	–	GU376558
*Maxonia apiifolia* (Sw.) C.Chr.	KJ464477	GU376709	KJ464641
*Megalastrum abundans* (Rosenst.) A.R.Sm. & R.C.Moran	KJ464478	–	KJ464642
*Megalastrum atrogriseum* (C.Chr.) A.R.Sm. & R.C.Moran	KJ464479	GU376710	KJ464643
*Megalastrum connexum* (Kaulf.) A.R.Sm. & R.C.Moran	KJ464481	–	KJ464645
*Megalastrum lanatum* (Fée) Holttum	KJ464483	–	KJ464647
*Megalastrum littorale* R.C.Moran, J.Prado & Labiak	–	GU376651	GU376561
*Megalastrum macrotheca* (Fée) A.R.Sm. & R.C.Moran	KJ464484	GU376697	KJ464648
*Megalastrum vastum* (Kunze) A.R.Sm. & R.C.Moran	KJ464487	GU376658	KJ464651
*Mickelia bernoullii* (Kuhn ex Christ) R.C.Moran, Labiak & Sundue	–	GU376666	GU376506
*Mickelia guianensis* (Aubl.) R.C.Moran, Labiak & Sundue	–	GU376667	GU376548
*Mickelia hemiotis* (Maxon) R.C.Moran, Labiak & Sundue	–	–	GU376512
*Mickelia nicotianifolia* (Sw.) R.C.Moran, Labiak & Sundue	–	KF667557	GU376519
*Mickelia oligarchica* (Baker) R.C.Moran, Labiak & Sundue	KJ464489	–	GU376520
*Mickelia scandens* (Raddi) R.C. Moran, Labiak & Sundue	–	GU376696	GU376547
*Olfersia cervina* Kunze	KJ464493	DQ153079	KJ464652
*Parapolystichum acuminatum* (Houlston) Labiak, Sundue & R.C.Moran	KJ464430	KC977454	KJ464601
*Parapolystichum boivinii* (Baker) Rouhan	KJ464435	–	KJ464607
*Parapolystichum confine* (Maxon ex C.Chr.) Labiak, Sundue & R.C.Moran	KJ464438	–	–
*Parapolystichum effusum* (Sw.) Ching	KJ464441	–	–
Parapolystichum effusum (Sw.) Ching subsp. divergens (Willd. ex Schkuhr) Tindale	KJ464440	–	–
*Parapolystichum excultum* (Mett.) Labiak, Sundue & R.C.Moran	–	KF709501	GU376541
*Parapolystichum glabellum* (A.Cunn.) Labiak, Sundue & R.C.Moran	KJ464445	KF709503	KJ464613
*Parapolystichum microsorum* (Endl.) Labiak, Sundue & R.C.Moran	KJ464451	GU376712	KJ464617
*Parapolystichum perrierianum* (C.Chr.) Rouhan	KJ464455	–	KJ464623
*Parapolystichum rufescens* (Blume) Labiak, Sundue & R.C.Moran	KJ464461	–	KJ464629
*Parapolystichum vogelii* (Hook.) Rouhan	KJ464470		
*Parapolystichum windsorensis* (D.L.Jones & B.Gray) Labiak, Sundue & R.C.Moran	KJ464473	–	KJ464639
*Pleocnemia conjugata* C.Presl	–	GU376713	KF709510
*Pleocnemia cumingiana* C.Presl	KJ196828	–	KJ196705
*Pleocnemia dahlii* (Hieron.) Holttum	KJ196829	–	KJ196706
*Pleocnemia hemiteliiformis* (Racib.) Holttum	KF709482	KF667560	KF709511
*Pleocnemia irregularis* (C.Presl) Holttum	KF709491	–	KF709513
*Pleocnemia leuzeana* (Gaudich.) C.Presl	KJ196830	–	–
*Pleocnemia olivacea* (Copel.) Holttum	KJ464495	–	–
*Pleocnemia presliana* Holttum	KJ464496	KF667561	–
*Pleocnemia rufinervis* Nakai	JF303976	KF667562	–
*Pleocnemia winitii* Holttum	EF460686	–	KF709515
*Polybotrya alfredii* Brade	KJ464497	KF667563	KJ464653
*Polybotrya andina* C.Chr.	KJ464498	KP271084	KJ464654
*Polybotrya pubens* Mart.	KJ464499	KP271085	–
Polystichum tsus-simense (Hook.) J.Sm. var. mayebarae (Tagawa) Sa.Kurata	AB575224	–	DQ150408
*Pseudotectaria biformis* (Mett.) Holttum	–	–	KF897951
*Pseudotectaria decaryana* (C.Chr.) Tardieu	–	–	KF897952
*Rumohra adiantiformis* (G.Forst.) Ching	KJ464500	–	KJ464655
*Rumohra berteroana* (Colla) J.J. Rodr.	KJ464503	–	KJ464657
*Stigmatopteris ichthiosma* (Sodiro) C.Chr.	KJ464504	–	KJ464658
*Stigmatopteris killipiana* Lellinger	KJ464505	–	KJ464659
*Stigmatopteris lechleri* (Mett) C.Chr.	KJ464506	KP271087	KJ464660
*Stigmatopteris sordida* (Maxon) C.Chr.	KJ464507	–	KJ464661
*Teratophyllum koordersii* Holttum	–	–	GU376566
*Teratophyllum ludens* (Fée) Holttum	–	–	GU376567
*Teratophyllum wilkesianum* Holttum	KJ464508	–	–
Nephrolepidaceae			
*Nephrolepis abrupta* (Bory) Mett.	HM748137	KF667559	–
*Nephrolepis acutifolia* (Desv.) Christ.	HM748139	–	–
*Nephrolepis biserrata* (Sw.) Schott	AB575227	GU376688	–
*Nephrolepis brownii* (Desv.) Hovenkamp & Miyam.	KR816691	–	–
*Nephrolepis cordifolia* (L.) C.Presl	AB575228	–	–
*Nephrolepis davalliae* Alderw.	HM748147	–	–
*Nephrolepis davallioides* Kunze	HM748148	GU376690	–
*Nephrolepis exaltata* (L.) Schott	HM748149	–	–
*Nephrolepis falcata* (Cav.) C.Chr.	HM748150	–	–
*Nephrolepis falciformis* J.Sm.	AB232404	–	–
*Nephrolepis lauterbachii* (Christ) Christ	HM748153	–	–
*Nephrolepis pectinata* (Willd.) Schott	HM748155	–	–
*Nephrolepis pendula* (Raddi) J.Sm.	HM748156	–	–
*Nephrolepis radicans* (Burm.) Kuhn	HM748157	–	–
*Nephrolepis rivularis* (Vahl) Mett.	HM748158	–	–
*Nephrolepis undulata* J.Sm.	HM748159	–	–
Lomariopsidaceae			
*Cyclopeltis crenata* (Fée) C.Chr.	DQ054517	EF540718	DQ51448
*Cyclopeltis novoguineensis* Rosenst.	**KY397974**	**KY397978**	**KY397970**
*Cyclopeltis semicordata* (Sw.) J.Sm.	EF463234	**KY397977**	**KY397969**
*Dracoglossum plantagineum* (Jacq.) Christenh.	KC914564	**KY397979**	**KY397971**
*Dracoglossum sinuatum* (Fée) Christenh.	–	–	KU605106
*Dryopolystichum phaeostigma* (Ces.) Copel.	**KY397972**	**KY397976**	**KY397968**
*Lomariopsis crassifolia* Holttum	–	–	DQ396559
*Lomariopsis guineensis* (Underw.) Alston	–	KJ628952	DQ396560
*Lomariopsis hederacea* Alston	–	–	DQ396561
*Lomariopsis jamaicensis* (Underw.) Holttum	–	–	DQ396562
*Lomariopsis japurensis* (C.Martius) J.Sm.	–	–	DQ396563
*Lomariopsis kunzeana* (Underw.) Holttum	–	–	DQ396569
*Lomariopsis latipinna* Stolze	–	–	DQ396571
*Lomariopsis lineata* (C.Presl) Holttum	–	–	DQ396572
*Lomariopsis longicaudata* (Bonap.) Holttum	–	–	Q396573
*Lomariopsis madagascarica* (Bonap.) Alston	–	–	DQ396575
*Lomariopsis mannii* (Underw.) Alston	–	–	DQ396577
*Lomariopsis marginata* (Schrad.) Kuhn	AY818677	–	DQ396578
*Lomariopsis maxonii* (Underw.) Holttum	–	–	DQ396580
*Lomariopsis muriculata* Holttum	–	–	DQ396582
*Lomariopsis palustris* (Hook.) Mett. ex Kuhn	–	HM748162	DQ396585
*Lomariopsis pervillei* Kuhn	–	–	DQ396586
*Lomariopsis pollicina* (Willemet) Mett. ex Kuhn	EF463235	–	DQ396588
*Lomariopsis prieuriana* Fée	–	–	DQ396590
*Lomariopsis recurvata* Fée	–	–	DQ396592
*Lomariopsis rossii* Holttum	–	–	DQ396594
*Lomariopsis salicifolia* (Kunze) Lellinger	–	–	DQ396595
*Lomariopsis sorbifolia* (L.) Fée	EF463236	–	–
*Lomariopsis spectabilis* Mett.	AB232401	–	KJ196685
*Lomariopsis vestita* E.Fourn.	–	–	DQ396598
*Lomariopsis wrightii* Mett.	–	–	DQ396600
Tectariaceae			
*Arthropteris altescandens* J.Sm.	KF667636	KF667550	KF667606
*Arthropteris articulata* (Brack.) C.Chr.	KC977367	KC977437	KC977411
*Arthropteris beckleri* (Hook.) Mett.	U05605	–	KF667607
*Arthropteris cameroonensis* Alston	KF667638	–	–
*Arthropteris guinanensis* H.G.Zhou & Y.Y.Huang	KC977364	KC977442	KC977404
*Arthropteris monocarpa* (Cordem.) C.Chr.	HM748132	–	KF897941
*Arthropteris orientalis* (Gmel.) Posth.	HM748133	KC977435	KC977420
*Arthropteris palisotii* (Desv.) Alston	AB575230	KC977427	KC977406
*Arthropteris parallela* (Baker) C.Chr.	EF463266	KC977453	KC977425
*Arthropteris paucivenia* (C.Chr.) H.M.Liu, Hovenkamp & H.Schneid.	EF463268	–	KC977426
*Arthropteris repens* (Brack.) C.Chr.	KC977368	KC977438	KC977412
*Arthropteris tenella* (G.Forst.) J.Sm. ex Hook.f.	KC977363	KF011547	KC977424
*Hypoderris brauniana* (H.Karst.) F.G.Wang & Christenh.	KF667647	–	KF667618
*Hypoderris brownii* J.Sm.	KF667642	–	KF667611
*Hypoderris nicotianifolia* (Baker) R.C.Moran, Labiak & J.Prado	KF667653	–	KF667626
*Pteridrys australis* Ching	KJ196892	–	KJ196678
*Pteridrys cnemidaria* (Christ) C.Chr. & Ching	KF709488	–	KF709517
*Pteridrys lofouensis* (Christ) C.Chr. & Ching	EF460687	KF667566	–
*Pteridrys microthecia* (Fée) C.Chr. & Ching	KJ196848	–	KF709518
*Pteridrys syrmatica* (Willd.) C.Chr. & Ching	KJ196875	–	KF709519
*Tectaria acerifolia* R.C.Moran	KF887170	–	KF897954
*Tectaria angulata* (Willd.) Copel.	KJ196876	–	KJ196656
*Tectaria aurita* (Sw.) S.Chandra	KJ196849	–	KJ196631
*Tectaria barberi* (Hook.) Copel.	KJ196846	–	KJ196628
*Tectaria borneensis* S.Y.Dong	KJ196854	KF667555	KJ196642
*Tectaria cicutaria* (L.) Copel.	KF667649	–	KF667620
*Tectaria coadunata* (J.Sm.) C.Chr.	KJ196851	–	KJ196661
*Tectaria crenata* Cav.	KF667650	KF667568	KF667621
*Tectaria decurrens* (C.Presl) Copel.	AB575232	–	DQ514524
*Tectaria devexa* (Kunze ex Mett.) Copel.	AB575233	KP271088	KF897956
*Tectaria dilacerata* (Kunze) Maxon	KF887173	–	KF897957
*Tectaria fauriei* Tagawa	AB575234	–	KJ196658
*Tectaria fernandensis* C.Chr.	KF887174	–	KF897958
*Tectaria gigantea* (Blume) Copel.	KJ196853	–	KJ196660
*Tectaria griffithii* (Baker) Ching	KF667652	–	KF667624
*Tectaria grossedentata* Ching & Chu H.Wang	KJ196882	KP271089	KJ196667
*Tectaria harlandii* (Hook.) C.M.Kuo	AB575231	–	KJ196648
*Tectaria harlandii* (Hook.) C.M.Kuo	KF887178	–	KF897961
*Tectaria heracleifolia* (Willd.) Underw.	KF887180	–	KF897963
*Tectaria herpetocaulos* Ching & Chu H. Wang	KJ196884	–	KJ196669
*Tectaria heterocarpa* C.V.Morton	KF887181	–	KF897964
*Tectaria impressa* (Fée) Holttum	KJ196841	–	KF897965
*Tectaria kusukusensis* (Hayata) Lellinger	EF460681	–	KF897968
*Tectaria labrusca* (Hook.) Copel.	KJ196818	–	KJ196692
*Tectaria luchunensis* S.K.Wu	KJ196845	KP271090	KJ196627
*Tectaria macleanii* (Copel.) S.Y.Dong	KJ196810	–	KJ196680
*Tectaria melanocaula* (Blume) Copel.	KJ196832	–	KJ196709
*Tectaria morsei* (Baker) P.J.Edwards ex S.Y.Dong	KJ196893	KF667570	KF561675
*Tectaria nayarii* Mazumdar	EF463267	–	KJ196699
*Tectaria paradoxa* (Fée) Sledge	KF887189	–	KF897971
*Tectaria phaeocaulis* (Rosenst.) C.Chr.	AB232397	KF709499	KF897972
*Tectaria pica* (L.) C.Chr.	KF887191	GU376715	KF897973
*Tectaria polymorpha* (Wall. ex Hook.) Copel.	KJ196888	GU376716	KJ196657
*Tectaria prolifera* (Hook.) R.M.Tryon & A.F.Tryon	EF463273	–	KF897974
*Tectaria psomiocarpa* S.Y.Dong	KJ196822	KF667572	KJ196698
*Tectaria pubens* R.C.Moran	KF887193	KF667573	KF897975
*Tectaria quinquefida* (Baker) Ching	KJ196885	–	KJ396622
*Tectaria repanda* (Willd.) Holttum	KJ196831	–	KJ196707
*Tectaria sagenioides* (Mett.) Christenh.	KF887194	KF667575	KF561672
*Tectaria semipinnata* (Roxb.) Morton	KJ196817	KF667577	KJ196691
*Tectaria simonsii* (Baker) Ching	AB575236	–	KF897977
*Tectaria singaporiana* (Wall. ex Hook. & Grev.) Ching	KF887196	–	KF897978
*Tectaria subglabra* (Holttum) S.Y.Dong	–	–	KJ196676
*Tectaria subsageniacea* (Christ) Christenh.	KF887197	KF667576	KF561670
*Tectaria subtriphylla* (Hook. & Arn.) Copel.	AB575237	–	KF897980
*Tectaria tricuspis* (Bedd.) Copel.	KJ196820	–	KJ196694
*Tectaria variolosa* (Wall. ex Hook.) C.Chr.	EF460690	–	KF897982
*Tectaria vasta* (Blume) Copel.	KF667655	–	KF667628
*Tectaria vivipara* Jermy & T.G.Walker	KF887201	–	KF897983
*Tectaria zeilanica* (Houtt.) Sledge	AB232395	–	KF709521
*Triplophyllum crassifolium* Holttum	KF887203	–	KF897985
*Triplophyllum fraternum* (Mett.) Holttum	KF667657	–	KF667630
*Triplophyllum funestum* (Kunze) Holttum	EF463276	–	KF667631
*Triplophyllum glabrum* J.Prado & R.C.Moran	KF887207	–	KF897989
*Triplophyllum heudelotii* Pic.Serm.	–	–	KF897990
*Triplophyllum jenseniae* (C.Chr.) Holttum	KF667660	–	KF667633
*Triplophyllum pentagonum* (Bonap.) Holttum	KF667662	–	KF667635
*Triplophyllum pilosissimum* (J.Sm. ex T.Moore) Holttum	–	–	KU605127
*Triplophyllum securidiforme* (Hook.) Holttum	–	–	KU605128
*Triplophyllum vogelii* (Hook.) Holttum	KF667661	–	KF667634
Oleandraceae			
*Oleandra articulata* (Sw.) C.Presl	KF667644	KF709500	KF667613
*Oleandra cumingii* J.Sm.	KJ196816	–	KJ196690
*Oleandra neriiformis* Cav.	KJ196815	–	KJ196689
*Oleandra pilosa* Hook.	KF667646	–	KF667615
Davalliaceae			
*Davallodes hirsuta* (J.Sm.) Copel.	AY096196	–	–
*Davallodes yunnanensis* (Christ) M.Kato & Tsutsumi	JX103718	KC914565	–
Polypodiaceae			
*Campyloneurum minus* Fée	KF667665	–	–
*Microgramma lycopodioides* (L.) Copel.	KF667664	–	–
*Niphidium longifolium* (Cav.) C.V.Morton & Lellinger	KF667663	KF709495	–

## Supplementary materials

Supplementary material 1 Figure S1. Maximum likelihood phylogram of Polypodiineae obtained from the combined (*rbcL + rps4-trnS + trnL-F*) dataset. Data type: statistical data Explanation note: Maximum likelihood bootstrap percentages (BS) are provided at each node. Thickened lines indicate Bayesian inference posterior probability (PP) ≥ 0.9. This dataset is made available under the Open Database License (http://opendatacommons.org/licenses/odbl/1.0/). The Open Database License (ODbL) is a license agreement intended to allow users to freely share, modify, and use this Dataset while maintaining this same freedom for others, provided that the original source and author(s) are credited.Cheng-Wei Chen, Michael Sundue, Li-Yaung Kuo, Wei-Chih Teng, Yao-Moan Huang

Supplementary material 2 Figure S2. Maximum likelihood phylogram of Polypodiineae obtained from the *rbcL* dataset. Data type: statistical data Explanation note: Maximum likelihood bootstrap percentages (BS) are provided at each node. Thickened lines indicate Bayesian inference posterior probability (PP) ≥ 0.9. This dataset is made available under the Open Database License (http://opendatacommons.org/licenses/odbl/1.0/). The Open Database License (ODbL) is a license agreement intended to allow users to freely share, modify, and use this Dataset while maintaining this same freedom for others, provided that the original source and author(s) are credited.Cheng-Wei Chen, Michael Sundue, Li-Yaung Kuo, Wei-Chih Teng, Yao-Moan Huang

Supplementary material 3 Figure S3. Maximum likelihood phylogram of Polypodiineae obtained from the *rps4-trnS* dataset. Data type: statistical data Explanation note: Maximum likelihood phylogram of Polypodiineae obtained from the *rps4-trnS* dataset. Maximum likelihood bootstrap percentages (BS) are provided at each node. Thickened lines indicate Bayesian inference posterior probability (PP) ≥ 0.9. This dataset is made available under the Open Database License (http://opendatacommons.org/licenses/odbl/1.0/). The Open Database License (ODbL) is a license agreement intended to allow users to freely share, modify, and use this Dataset while maintaining this same freedom for others, provided that the original source and author(s) are credited.Cheng-Wei Chen, Michael Sundue, Li-Yaung Kuo, Wei-Chih Teng, Yao-Moan Huang

Supplementary material 4 Figure S4. Maximum likelihood phylogram of Polypodiineae obtained from the *trnL-F* dataset. Data type: statistical data Explanation note: Maximum likelihood bootstrap percentages (BS) are provided at each node. Thickened lines indicate Bayesian inference posterior probability (PP) ≥ 0.9. This dataset is made available under the Open Database License (http://opendatacommons.org/licenses/odbl/1.0/). The Open Database License (ODbL) is a license agreement intended to allow users to freely share, modify, and use this Dataset while maintaining this same freedom for others, provided that the original source and author(s) are credited.Cheng-Wei Chen, Michael Sundue, Li-Yaung Kuo, Wei-Chih Teng, Yao-Moan Huang
