# Supplementary figures and images for: Phylogenetic analyses place the monotypic Dryopolystichum within Lomariopsidaceae
Source: PhytoKeys. 2017 Apr 7;(78):83–107. doi: 10.3897/phytokeys.78.12040 (PMC5543276; doi:10.3897/phytokeys.78.12040)

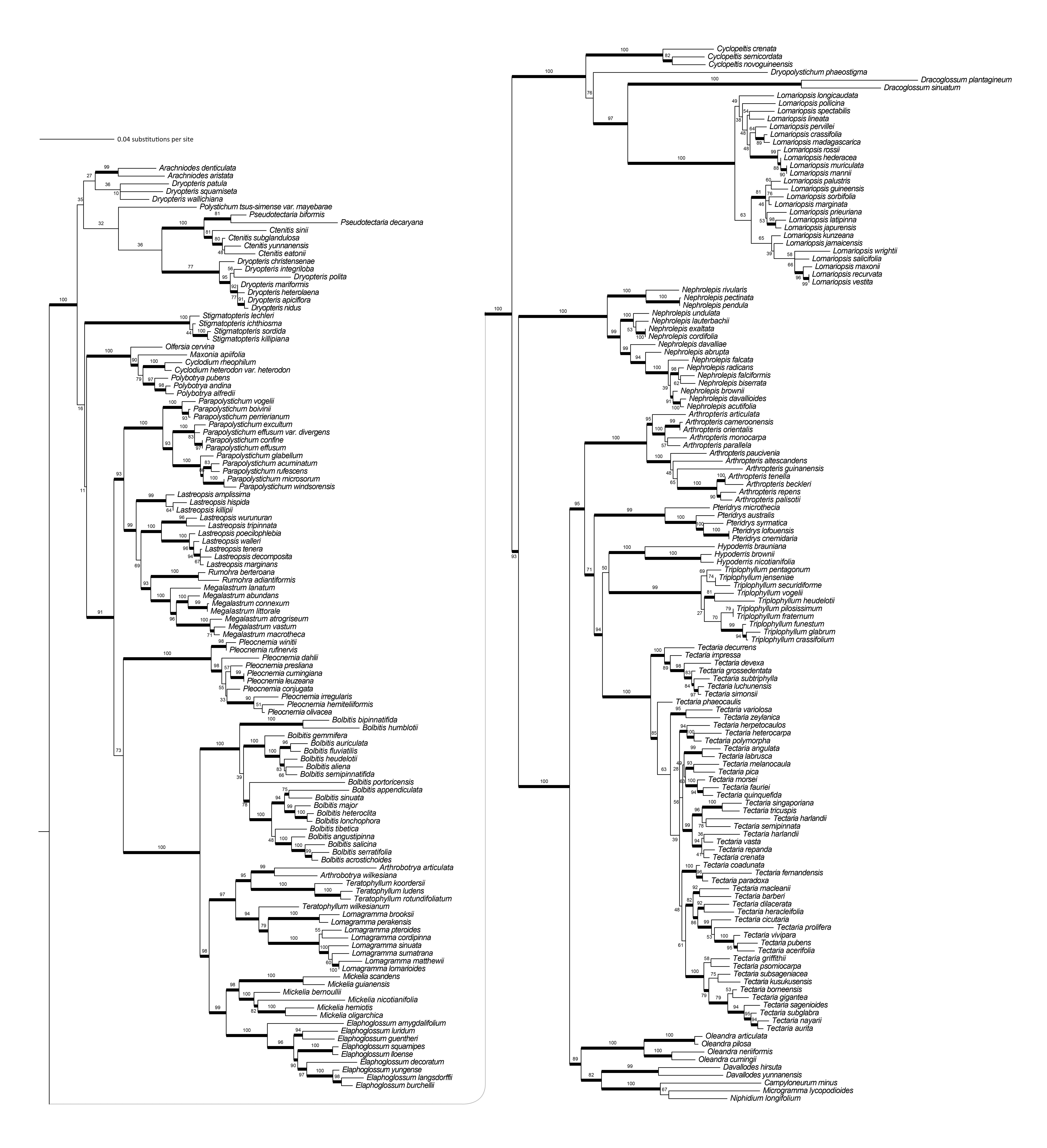

Supplement: Supplementary material 1 — Figure S1. Maximum likelihood phylogram of Polypodiineae obtained from the combined (rbcL + rps4-trnS + trnL-F) dataset. [file phytokeys-78-e12040-s001.jpg]

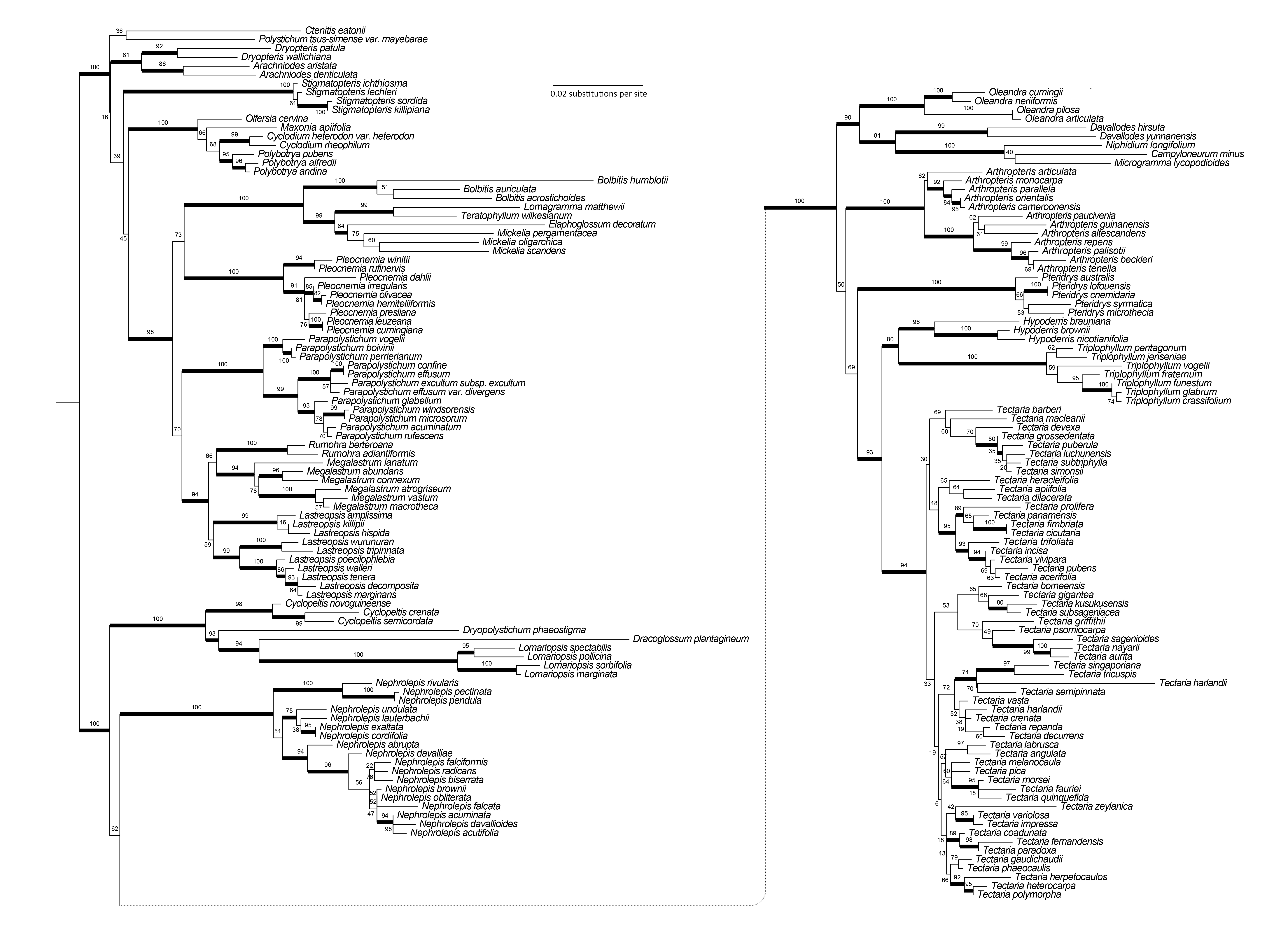

Supplement: Supplementary material 2 — Figure S2. Maximum likelihood phylogram of Polypodiineae obtained from the rbcL dataset. [file phytokeys-78-e12040-s002.jpg]

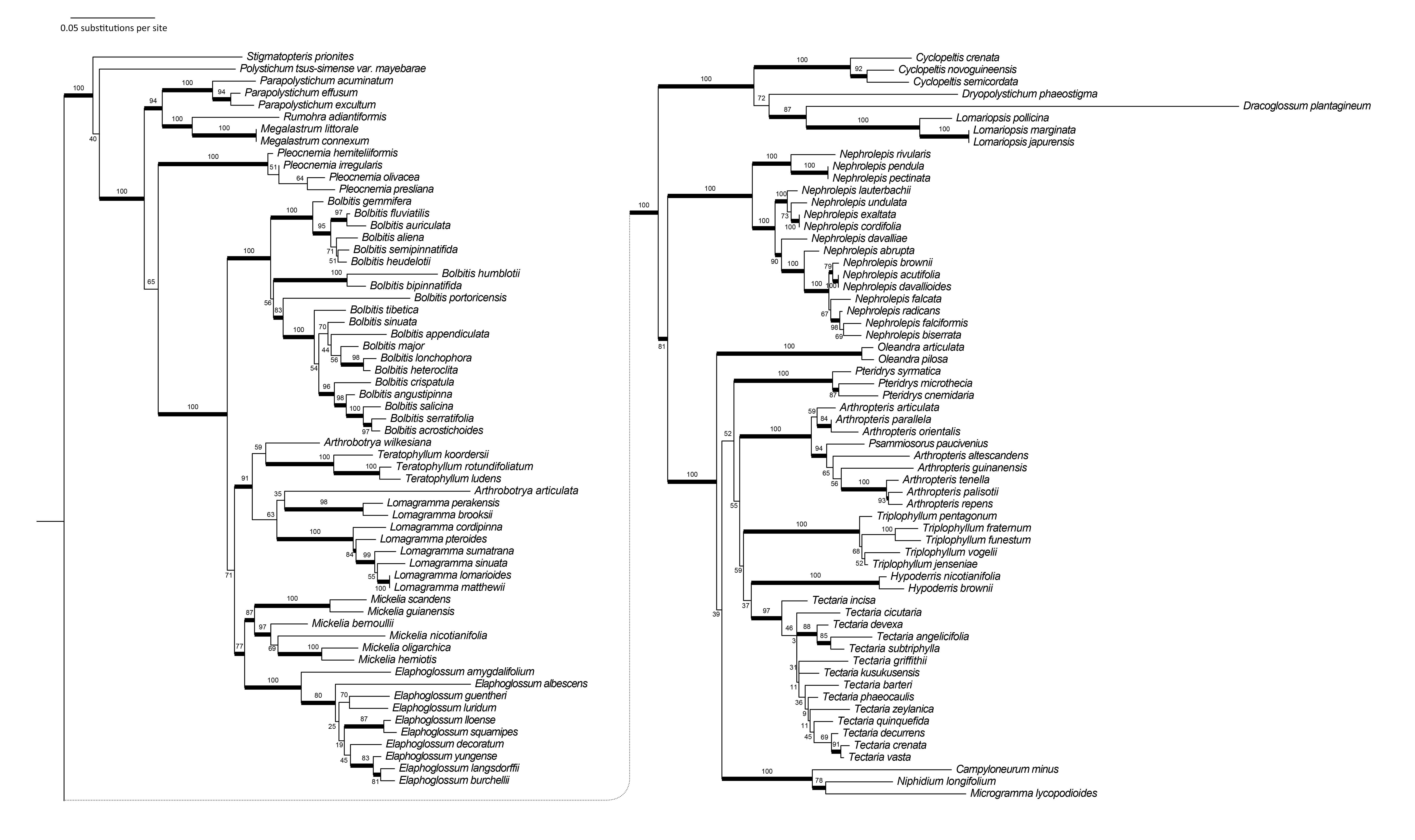

Supplement: Supplementary material 3 — Figure S3. Maximum likelihood phylogram of Polypodiineae obtained from the rps4-trnS dataset. [file phytokeys-78-e12040-s003.jpg]

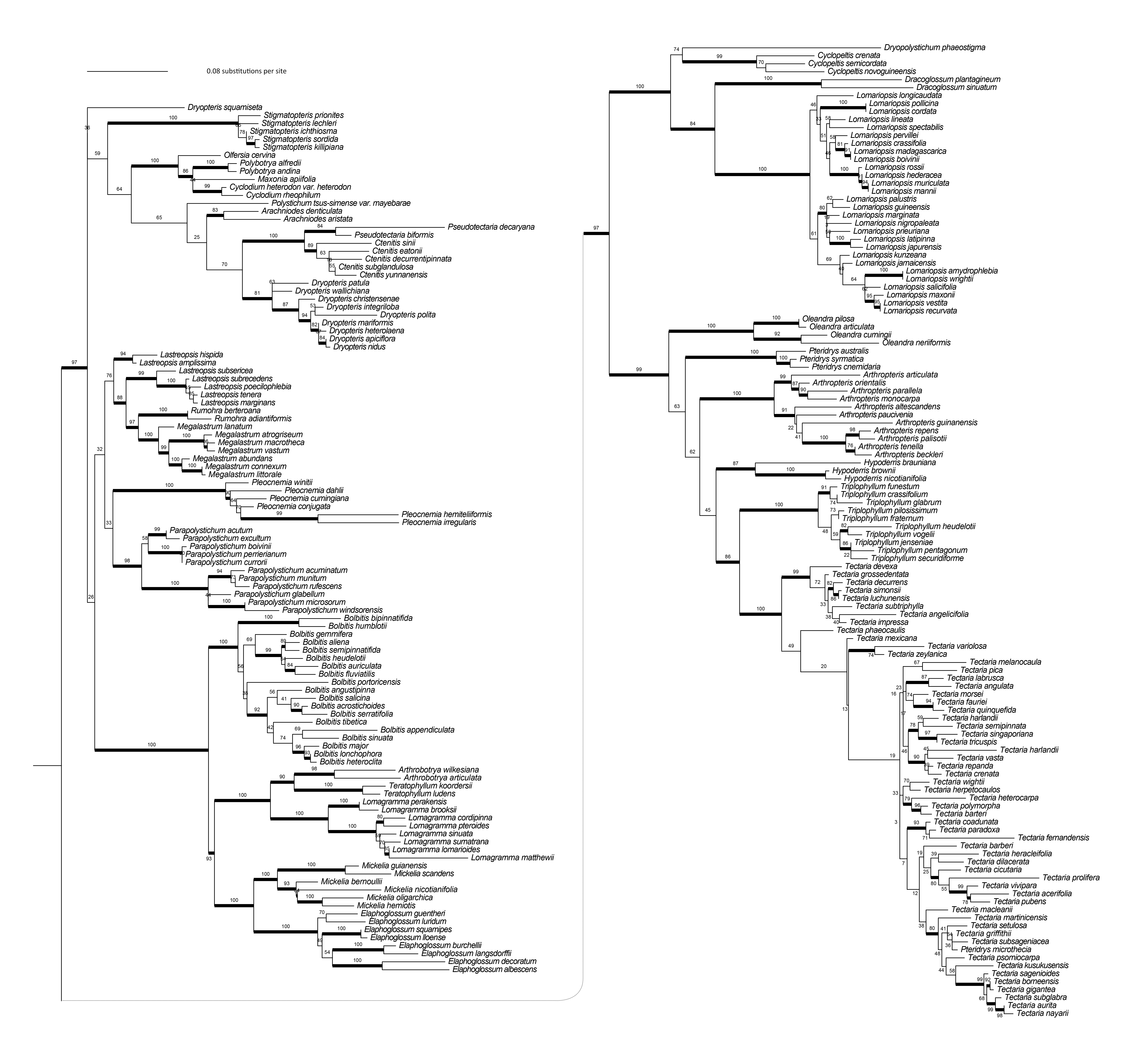

Supplement: Supplementary material 4 — Figure S4. Maximum likelihood phylogram of Polypodiineae obtained from the trnL-F dataset. [file phytokeys-78-e12040-s004.jpg]
